# Informed consent in clinical practice: patients’ experiences and perspectives following surgery

**DOI:** 10.1186/s13104-015-1754-z

**Published:** 2015-12-09

**Authors:** Joseph Ochieng, William Buwembo, Ian Munabi, Charles Ibingira, Haruna Kiryowa, Gabriel Nzarubara, Erisa Mwaka

**Affiliations:** Anatomy Department, School of Biomedical Sciences, Makerere University, P.O 7072, Kampala, Uganda; Anatomy Department, St. Augustine International University, Kampala, Uganda

**Keywords:** Patients, Perspectives, Experience, Informed consent, Surgery

## Abstract

**Background:**

Informed consent during medical practice is an essential component of comprehensive medical care and is a requirement that should be sought all the time the doctor interacts with the patients, though very challenging when it comes to implementation. Since the magnitude and frequency of surgery related risk are higher in a resource limited setting, informed consent for surgery in such settings should be more comprehensive. This study set out to evaluate patients’ experiences and perspectives of informed consent for surgery.

**Methods:**

This was a survey of post-operative patients at three university teaching hospitals in Uganda. The participants were interviewed using guided, semi-structured questionnaires. Patients from different surgical disciplines participated in the study.

**Results:**

A total of 371 patients participated in the study. Eighty percent of the participants reported having been given explanations on the indication for their surgery, 56.1 % had all their questions answered before the operation, 17 % did not know the type of operation they had undergone and another 17 % did not give their consent for the operation. Additionally, more than 81 % of the participants reported giving their own permission for surgery, although only 23.7 % were able to identify the person who obtained consent from them and 22.4 % knew the names of the surgeons who conducted the surgical procedure on them. About 20 % of the participants were not satisfied with the information provided by both the doctor before and after the operation. However, there were varying responses on when doctors should explain to patients with the majority saying it should be done before treatment or surgery, while others thought it should be done on admission, others proposed that it be made immediately after the examination among other responses. On what should be done to improve communication between doctors and patients, a number of suggestions, including the need for a detailed explanation for the patient by the doctor about their disease conditions and treatment options were suggested.

**Conclusions:**

Patients’ perceptions of what constitutes informed consent are diverse and many patients undergo surgery without knowledge of the identity of the surgeon or the reason for the surgery. There is a need to improve on patients’ participation in informed decision making, and this can be achieved through continuing medical education for doctors.

## Background

Informed consent during medical practice is an essential component of comprehensive medical care and is a requirement that should be sought all the time doctor interacts with the patients, although it is very challenging when it comes to implementation [1–11]. Effective administration, comprehension and documentation of informed consent for medical care is key to a healthy doctor-patient relationship since it highlights respect of the patients’ rights [4, 5].

The extent of detail of informed consent varies with the magnitude of the anticipated risk and thus informed consent for surgery is expected to be more detailed as compared to general medical care. Since the magnitude and frequency of surgery related risk is even higher in a resource limited settings, informed consent for surgery in such settings should be more detailed [8, 12]. Despite the high disease burden and high risks associated with surgical care, to our knowledge, no formal evaluation of what surgical patients go through in terms of participation in decision making had been conducted in our multi-cultural low resource setting. The study to highlight unique aspects of informed consent in our setting was important [12]. This study set out to describe an evaluation of patient’s experiences and perspectives on informed consent for surgery.

## Methods

This was a survey at three university teaching hospitals in Uganda that recruited 371 postoperative patients. Only patients 18 years of age and above participated in the study and were recruited from general surgery, orthopedic surgery, Otorhinolaryngology (Ear-Nose-Throat), ophthalmology, dentistry, obstetrics and gynaecology surgical units. Participants were interviewed within 2 weeks of surgery using an interviewer guided semi-structured questionnaire adopted from a previous study [13]. The study variables included: patient demographic data, duration of stay in the hospital, involvement in the decision making, adequacy of participant participation, satisfaction with the decision making process and any suggested improvements as summarized in Tables 1, 2. The data were entered into Epidata version 3.2 (Epidata association, Denmark) and exported to SPSS 17 where after checking for duplicate entries a preliminary analysis of each variable was made to identify the additional range and omission errors. Averages for questions that required quantitative answers and frequency tables for questions that required a choice among several given alternatives were calculated, bar graphs and pie charts were constructed. For the adjusted ORs, multi-variable regression analysis was done.

**Table 1:** Descriptive statistics of the participant population

Item	Number	Mean (median)	SD (inter quartile range)	OR (95 % CI)
Age	371	31.78	12.32	1.04 (1.01–1.07)
Sex^a^	371	0.49	0.50	1.13 (0.66–1.95)
Education	366	(2)	(0–4)	1.03 (0.74–1.45)
Duration of stay in hospital (days)	371	18.43	38.35	1 (0.99–1.01)
Knew the type of operation they underwent^a^	370	0.85	0.36	2.29 (1.17–4.51)
Had all their questions answered before the operation^a^	362	0.57	0.50	11.06 (5.23–23.35)
Gave their own permission for the operation^a^	366	0.83	0.38	1
Knew the name of the surgeon that obtained their permission for the operation^a^	366	0.24	0.43	2.44 (1.11–5.36)
Knew the name of the surgeon that operated^a^	365	0.23	0.42	1.66 (0.81–3.44)
Was satisfied with the information provided^a^	367	0.78	0.42	4.38 (2.43–7.89)
Agreed on necessity of doctors to provide full details of disease management^a^	367	0.98	0.13	5.05 (1.00–25.64)

^a^Items coded as 1, and 0

**Table 2:** Adjusted odds ratio for the study model on adequate consent

Item	OR (95 % CI)	Adjusted odds ratio (95 % CI)
Age	1.04 (1.01–1.07)	1.04 (1.00–1.06)
Sex	1.13 (0.66–1.95)	2.16 (1.04–4.47)
Education	1.03 (0.74–1.45)	1.20 (0.75–1.91)
Duration of stay in hospital (days)	1 (0.99–1.01)	0.99 (0.99–1.00)
Knew the type of operation they underwent	2.29 (1.17–4.51)	1.89 (0.79–4.50)
Had all their questions answered before the operation	11.06 (5.23–23.35)	8.43 (3.76–18.93)
Gave their own permission for the operation	1	1
Knew the name of the surgeon that obtained their permission for the operation	2.44 (1.11–5.36)	1.92 (0.59–6.64)
Knew the name of the surgeon that operated	1.66 (0.81–3.44)	0.74 (0.22–6.64)
Was satisfied with the information provided	4.38 (2.43–7.89)	1.97 (0.94–3.87)
Agreed on necessity of doctors to provide full details of disease management	5.05 (1.00–25.64)	3.41 (0.47–24.80)

For questions that attracted open ended answers, thematic and content analysis was carried out whereby field notes were categorized according to the research themes and interpreted in line with the study objectives and research questions. Relevant comparisons were made between the different groups of informants.

### Ethical considerations

Ethical review and approval was sought from the Makerere University School of Biomedical Sciences Research and Ethics Committee, and the Uganda National Council for Science and Technology. Permission to conduct the study was obtained from the respective hospital administrations. Informed consent was obtained before recruitment of any participant into the study. Participant’s identifying information was kept confidential.

## Results

The study enrolled 371; 50.7 % of the participants were female giving a female to male ratio of almost 1:1. The age range was 18–80 years with a mean age of 31.8 and SD 12.3. More than 47 % of the participants had at least attained secondary school education, while 37.1 % had primary education, giving a literacy level of more than 94 % as shown in Table 1. The average hospital stay was 18.4 days and all participants recruited in the study had undergone surgical operation within the previous 2 weeks.

Although 80 % of the participants admitted having been given explanations on the indication of their surgery only 56.1 % had all their questions answered before the operation, 17 % did not know the type of surgical procedure they had undergone while another 17 % did not give their consent for the operation. Additionally, more that 81 % of the participants reported giving their own permission for surgery, although only 23.7 % would name the person who obtained consent from them and 22.4 % knew the names of the surgeons who operated on them (Table 1). About 20 % of the participants were not satisfied with the information provided by both the doctor before and after the operation.

More than 80 % of the participants reported that their condition was explained to them before surgery. And while more than 98 % agreed that treatment should be well explained by the doctors to patients, 46 % of participants reported no issues discussed concerning their condition.

However, there were varying responses on time points when doctors should explain to patients with the majority saying it should be done before treatment or surgery, while others thought it should be done on admission, others proposed that it be made immediately after the examination among other answers.

On what should be done to improve communication between doctors and patients, a number of suggestions were given. These included; the need for a detailed explanation for the patient by the doctor about their disease conditions and treatment options; that doctors should be kinder, more courteous, gentle and should not be rude; that doctors are doing a good job keep it up; that doctors should be available to patients at all times.

In Table 1, the majority of the respondents (83 %) gave their own permission (consent) for the operation. It is also important to note in Table 1 that only: Age (OR 1.04, 95 % CI 1.01–1.07), knowing the type of operation they underwent (OR 2.29 95 % CI 1.17–4.51), having all their questions answered prior to the operation (OR 11.06 95 % CI 5.23–23.35), knowing the name of the surgeon that obtained their consent (OR 2.44 95 % CI 1.11–5.36) and being satisfied with the information provided about the operation (OR 4.38, 95 % CI 2.43–7.89), were found to have significant increases for their odds ratios on uni-variable analysis with respect to the individual giving their own permission (consent) for the operation.

In Table 2 for the adjusted odds ratios, note that all the variables used for the study were retained as directed by our model for adequate informed consent. On adjustment the following were not statistically significant with respect to the individual giving their informed consent for the operation: education, duration of stay in the hospital, knowledge of the type of operation, knowing the names of either the surgeon that obtained their consent or operated them, satisfaction with the information provided and, agreement on the need to provide details of the operation. There was a significant increase of 4 % per year in the odds of one giving their permission for the operation with respect to the respondents age (Adjusted OR 1.04 95 % CI 1.00–1.06). Male respondents were twice as likely to give their own permission for the operation, this was significant (adjusted OR 2.16 95 % CI 1.04–4.47). Respondents who had all their questions answered prior to the operation were eight-time more like to give their own permission for the operation, this too was significant (adjusted OR 8.43 95 % CI 3.76–18.93).

## Discussion

We set out to describe the patients’ views and share their experience on informed decision making following their recent surgery. We found that informed consent is perceived differently by different individuals and what is practiced in our setting is far below the optimal standard. The difference in perception of informed consent is expected to have risen from the diverse composition of participants who varied greatly in terms of age, education, region of the country and socioeconomic status. The findings of this study are similar to what was reported in a related study [13].

The participants represented a wide range of age groups which is reflective of surgical conditions in the country. Individuals of all age groups undergo surgical treatment in the setting where the research was conducted. Only adult patients were included in the study since the objective was to appreciate the informed consent experiences of autonomous individuals.

Participants in the study included individuals of all educational levels (Table 1) and from all different social classes, which is reflective of the fact that most of the university teaching hospitals in the country are actually public health units and attend to all types of patients irrespective of the socio-economic status. Interviews were conducted within 2 weeks following surgery and this was aimed at reducing the effect of recall bias.

Although more than 80 % of the participants reported that their conditions were explained, only 56.1 % had all their questions answered before the operation (Table 2). It is important that all surgical patients be explained to their satisfaction answering: what the surgery involves, possible benefits, the risks and complications during and after the operation as well as the expected quality of life in the short and long term [12].

The majority of participants (98.6 %) agreed that treatment should be well explained by the doctors to patients although, what needs to be addressed in our setting is basically to empower the patients with adequate information so that they can actively participate in the surgical decision making process. This is because patients hold their own perceptions of surgery and what constitutes significant risk based on personal values and beliefs [14].

Guidance on how to participate in decision making for surgical care has been tested in other places and can be useful in this setting as well [15].

About 17 % of the participants did not know the type of operation they had undergone which implies that many patients undergo surgery without giving informed consent or even knowing what type of operation they undergo. This is highlighted in a related study where many doctors were found not to obtain informed consent [13]. Doctors should be educated that informed consent is a patients’ right that should be respected and solicited for at all times when they interact with patients [16].

Although 23.7 % of the participants knew the identity of the person who obtained consent from them and 22.4 % named the doctor who operated on them (Table 1), yet an overwhelming 82 % believed that they had given their consent for surgery. This highlights the fact that patients in our setting are not conversant with what constitutes informed consent and are not aware of their rights as patients when it comes to decision making during medical practice. In this setting, it would be difficult to believe that patients consent was adequately solicited for when the most basic aspect of consent that patients usually appreciate is the doctors name even if all the other information is forgotten. There is need to develop a mechanism that would improve shared decision making [15].

Additionally, more that 20 % of the participants reported their lack of satisfaction to doctor’s explanations both before and after operation (Table 1). This 20 % dissatisfaction though seemed lower than expected because many surgeons in this setting do not actually obtain what would be considered adequate informed consent [13]. This needs to be urgently addressed to understand what patients prefer and avoid consequences of such dissatisfaction in the future [14]. There is need for continuing medical education to the doctors on the importance of a health doctor patient relationship in addition to provision of services where patients’ grievances can be addressed. The problem of doctors’ lack of knowledge and practice of medical ethics has been highlighted elsewhere [11, 13].

**Fig. 1:**
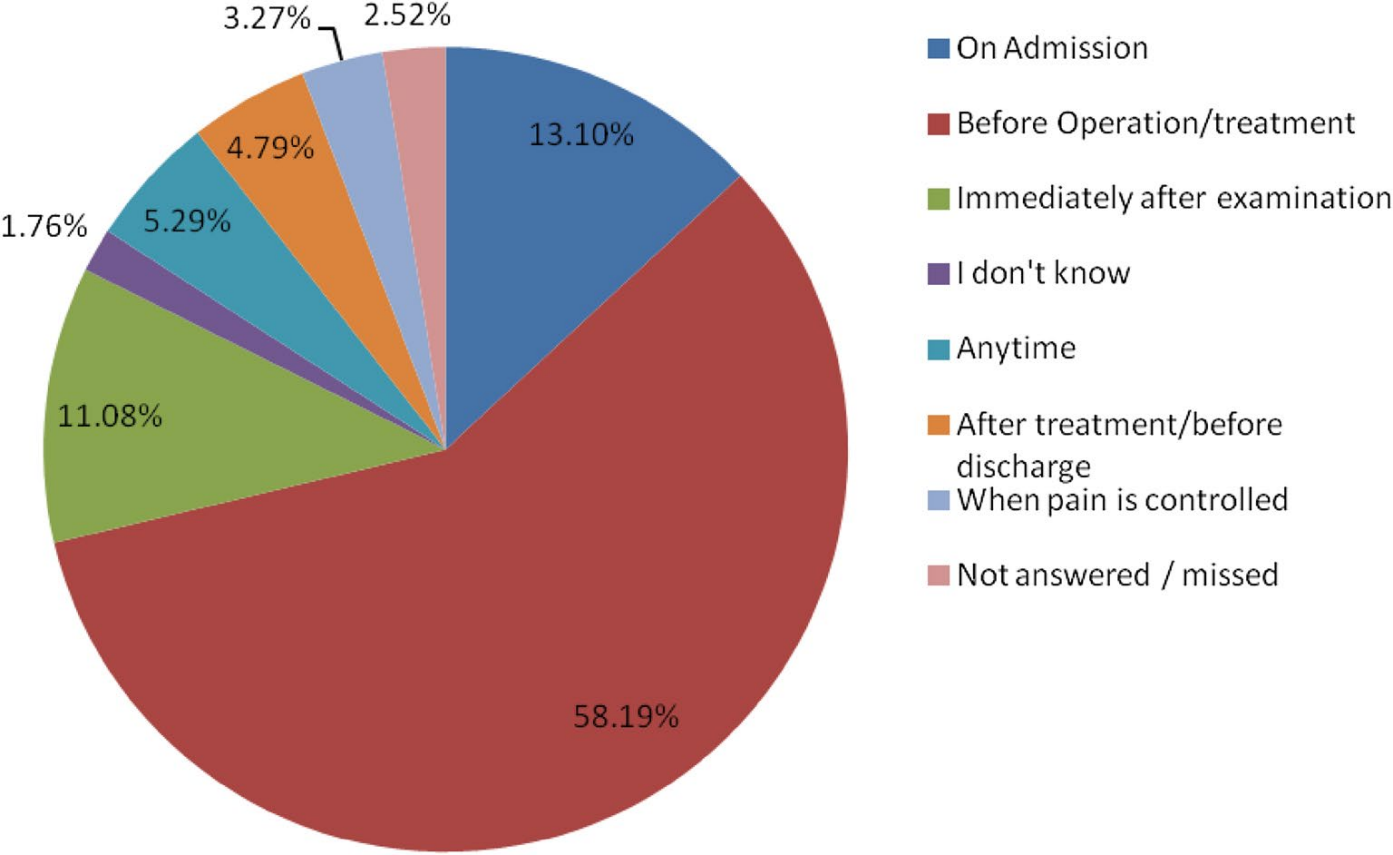
When consent should be sought

Despite the fact that more than 98 % of the participants agreed that treatment should be well explained by the doctors to patients, they were varying responses on what should be explained and when doctors should explain to patients with majority saying it should be done before treatment or surgery, while others thought it should be done on admission, others proposed immediately after examination and a number of other answers (Fig. 1). This needs to be stream lined by educating both the doctors and the patients on what constitutes informed consent which is a continuous process of information exchange and goes on all the time a doctor interacts with the patient [16].

The challenge of inadequate disclosure of information to patients during medical practice has been reported in other parts of the world where health care resources may not be as limited [14, 16, 17].

However, the challenges of informed consent in our setting are still magnificent given that most of the patients are ignorant about what constitutes informed decision making. This is highlighted by the fact that majority of participants could not identify the surgeons who obtained consent or performed the surgical procedure yet they still believed they gave their own permission for surgery.

## Limitations

The study was only conducted at university teaching hospitals which may not be reflective of what happens at other hospitals that are not associated with universities.

Since the university hospitals are the major referral hospitals in the country and host the most senior surgeons, the findings of this study may not highlight what happens at the lower none referral hospitals.

## Conclusions and recommendations

Patients’ perceptions of what constitutes informed consent are diverse and many undergo surgery without knowledge of the identity of the surgeon or the indication for surgery. In this setting, it would be difficult to believe that patients consent was adequately solicited for when the most basic aspect of consent that patients usually appreciate is the doctor’s name even if all the other information is forgotten. There is a need to improve on patients’ participation in informed decision making via continuing medical education for doctors.
